# Long noncoding RNA SNHG1 alleviates high glucose-induced vascular smooth muscle cells calcification/senescence by post-transcriptionally regulating Bhlhe40 and autophagy via Atg10

**DOI:** 10.1007/s13105-022-00924-2

**Published:** 2022-10-04

**Authors:** Shuang Li, Yuqing Ni, Chen Li, Qunyan Xiang, Yan Zhao, Hui Xu, Wu Huang, Yanjiao Wang, Yi Wang, Junkun Zhan, Youshuo Liu

**Affiliations:** Department of Geriatrics, Institute of Aging and Age-Related Disease Research, The Second Xiangya Hospital, Central SouthUniversity, Changsha, 410011 Hunan China

**Keywords:** Diabetic vascular calcification/aging, Long noncoding RNA, Bhlhe40, VSMCs, Autophagy

## Abstract

Long noncoding RNAs (lncRNAs) are emerging regulators of vascular diseases, yet their role in diabetic vascular calcification/aging remains poorly understood. In this study, we identified a down-expressed lncRNA SNHG1 in high glucose (HG)-induced vascular smooth muscle cells (HA-VSMCs), which induced excessive autophagy and promoted HA-VSMCs calcification/senescence. Overexpression of SNHG1 alleviated HG-induced HA-VSMCs calcification/senescence. The molecular mechanisms of SNHG1 in HA-VSMCs calcification/senescence were explored by RNA pull-down, RNA immunoprecipitation, RNA stability assay, luciferase reporter assay, immunoprecipitation and Western blot assays. In one mechanism, SNHG1 directly interacted with Bhlhe40 mRNA 3′-untranslated region and increased Bhlhe40 mRNA stability and expression. In another mechanism, SNHG1 enhanced Bhlhe40 protein SUMOylation by serving as a scaffold to facilitate the binding of SUMO E3 ligase PIAS3 and Bhlhe40 protein, resulting in increased nuclear translocation of Bhlhe40 protein. Moreover, Bhlhe40 suppressed the expression of Atg10, which is involved in the process of autophagosome formation. Collectively, the protective effect of SNHG1 on HG-induced HA-VSMCs calcification/senescence is accomplished by stabilizing Bhlhe40 mRNA and promoting the nuclear translocation of Bhlhe40 protein. Our study could provide a novel approach for diabetic vascular calcification/aging.

**Supplementary Information:**

The online version contains supplementary material available at 10.1007/s13105-022-00924-2.

## Introduction

Diabetes mellitus has become a global public health concern. According to the latest report, the overall prevalence of diabetes mellitus in adults is estimated to rise from 415 million in 2015 to 642 million by 2040 (Ogurtsova et al., [1]. These individuals have a twofold to threefold increased risk of cardiovascular disease (CVD) compared with those without diabetes mellitus [2]. In addition, CVD is considered as the leading cause for diabetes-related morbidity and mortality, imposing enormous social and financial burden with limited treatment options (Ogurtsova et al., [1]. Vascular calcification/aging is critical causative factors in the pathogenesis of CVD.

It is generally accepted that high glucose (HG)-induced vascular calcification/aging occurs in both the intimal and medial layers of the arteries [3, 4]. In particular, vascular smooth muscle cells (VSMCs), which locate in the tunica media, have key roles in vascular calcification/aging in response to HG condition [4]. However, little information exists regarding the cellular and molecular mechanisms underlying HG-induced VSMCs calcification/senescence.

Long noncoding RNAs (lncRNAs) are RNA transcripts longer than 200 nucleotides with limited or no protein-coding potential. Recent studies have identified a number of lncRNAs that play important roles in the development and progression of vascular diseases [5, 6]. LncRNA TUG1 is reported to regulate osteogenic differentiation in calcific aortic valve disease [6]. LncRNA CAIF attenuates myocardial infarction via inhibition of autophagy [5]. Although the aberrant expression and roles of these lncRNAs have been reported, it is likely that there are still more functional lncRNAs with involvement in diabetic vascular calcification/aging pathogenesis yet to be identified.

Small ubiquitin-related Modifier (SUMO) is a type of post-translational modification that can alter the protein activity, stability, interactions, and intracellular distribution [7]. SUMO protein consists of at least three isoforms–SUMO1, SUMO2, and SUMO3 [7]. The SUMO cycle consists of an E1 activating enzyme, an E2 conjugating enzyme, and an E3 protein ligase, in which the SUMO E3 ligase could facilitate SUMOylation [7].

In this study, a down-expressed lncRNA small nucleolar RNA host gene 1 (SNHG1) was identified in HG-induced calcified/senescent VSMCs. Recently, SNHG1 has been shown to be involved in many cancers [8], [9]. However, to date, the role of SNHG1 in diabetic vascular calcification/aging has not been explored yet. Functional experiments further revealed a regulator role of SNHG1 in transcription factor basic helix-loop-helix family member e40 (Bhlhe40) mRNA stability and expression via forming a protective RNA-RNA duplex with Bhlhe40 mRNA. Moreover, SNHG1 could bind with Bhlhe40 and PIAS3 protein, and enhance Bhlhe40 SUMOylation to facilitate the nuclear translocation of Bhlhe40 protein.

## Materials and methods

### Microarray data processing

Data retrieval was performed with the Gene Expression Omnibus (GEO, https://www.ncbi.nlm.nih.gov/geo/) database using “high glucose” and “VSMCs” as keywords. The gene expression profile of GSE17556 was downloaded. Since one dataset may not be as accurate as desired, “high glucose” and “HEK293” were used as keywords for further data collection. The gene expression profile of GSE15575 was downloaded. The two datasets were standardized datasets. GSE17556 included normal glucose (NG, 5 mM glucose)- and HG (25 mM glucose)-induced human aortic VSMCs (HA-VSMCs) while GSE15575 included NG- and HG-induced human embryonic kidney (HEK293) cells. We used GEO2R online software to analysis the raw submitter-supplied data of microarrays and identify differentially expressed lncRNAs between NG and HG conditions [10]. *P* < 0.05 and |log_2_FC|> 2 were used as the cut-off criteria to find differentially expressed lncRNAs.

### Cell culture

HA-VSMCs were obtained from ATCC (ATCC-CRL-1999). The cells were cultured in Dulbecco’s Modified Eagle’s Medium (DMEM; Hyclone, USA) with 10% fetal bovine serum (FBS; Invitrogen, USA), 100 U/mL penicillin, and 100 µg/mL streptomycin at 37℃ with 5% CO_2._ HA-VSMCs were cultured with NG (5 mM glucose) or HG (30 mM glucose) for 24 or 48 h. We changed the media every 2 days and passaged cells every 3–4 days.

HA-VSMCs were fixed in 2% formaldehyde and 0.2% glutaraldehyde for 10 min at room temperature and then washed with phosphate-buffered saline (PBS). VSMCs senescence was determined with senescence-associated β-galactosidase (SA-β-gal) Staining Kit (Beyotime Institute of Biotechnology, China) according to the manufacturer’s protocol. The SA-β-gal-positive staining was determined as previously described [4].

### Alizarin Red S staining

HA-VSMCs were subjected to different treatments. Then, Alizarin Red S staining was done as previously described [11].

### Transfection

PcDNA3.1-SNHG1 and its negative control were designed and synthesized by GenePharma Co. Ltd. (China). Si-SNHG1 (si-SNHG1-1 and i-SNHG1-2), si-Bhlhe40 (si-Bhlhe40-1, si-Bhlhe40-2, and si-Bhlhe40-3), and their negative controls (si-Con) were designed and synthesized by GenePharma Co. Ltd. (China). The sequences of si-SNHG1 and si-Bhlhe40 used in this study are shown in Supplementary Table 1. Adenovirus vectors were used for transient overexpression of Bhlhe40 and Atg10 (GenePharma Co. Ltd., China). K279R and K159R mutants were generated using the QuickChange Site-Directed Mutagenesis Kit (Stratagene, USA), following the manufacturer’s instruction. Primers used for site-directed mutagenesis were as follows: Bhlhe40 K279R, 5′-GTCAGCACAATTAGGCAAGAA TCCGAA-3′ and 5′-TTCGGATTCTTGCCTAATTGTGCTGAC-3′; Bhlhe40 K159R, 5′-CAGTACCTGGCGAGGCATGAGAACACT-3′ and 5′-AGTGTTCTCATGCCTCGCCAGGTACTG-3′. Transfection of plasmids, siRNAs or adenovirus vectors was performed using Lipofectamine 3000 Reagent (Invitrogen, USA) according to the manufacturer’s protocol. We detected RNA levels after transfecting plasmids, siRNAs or adenovirus vectors for 24 h.

### Isolation of RNAs from cytoplasm and nucleus

The separation of the nuclear and cytoplasmic RNAs was carried out by using the PARIS Kit (Invitrogen, USA) according to the manufacturer’s protocol.

### Quantitative real-time PCR

Total RNA was isolated from cultured HA-VSMCs using TRIzol Reagent (Invitrogen, USA). RNA was reverse transcribed using RevertAid H Minus First Strand cDNA Synthesis Kit (Fermentas, USA) and followed by quantitative RT-PCR labeled by SYBR Green PCR Master Mix (ABI, USA). Data were normalized to values for GAPDH. Primers for qRT-PCR are listed in Supplementary Table 2.

### Nuclear and cytoplasmic protein extraction

The cytoplasmic and nuclear extracts were separated and prepared from HA-VSMCs using NE-PER nuclear and cytoplasmic extraction reagents (Thermo Fisher Scientific, USA) according to the manufacturer’s protocol.

### Immunoprecipitation and Western blot assays

Immunoprecipitation and Western blot assays were performed according to the previously described procedures [4]. Antibodies Runx2 (Abcam, UK, 1:1000), ALP (Abcam, UK, 1:2000), SM22ɑ (Abcam, UK, 1:1000), p16 (Proteintech, USA, 1:500), p21 (Cell signaling, USA, 1:1000), LC3-II (Abcam, UK, 1:500), SQSTM1 (Abcam, UK, 1:1000), Atg10 (Abcam, UK, 1:1000), Bhlhe40 (Proteintech, USA, 1:500), GAPDH (Abcam, UK, 1:4000), and β-actin (Proteintech, USA, 1:2000) were used in this study. HA-VSMCs were transfected as indicated and lysed in an immunoprecipitation buffer. The lysates were centrifuged for 20 min. Supernatants were incubated with specific antibodies overnight at 4℃ and protein A/G Agarose beads for 4 h. The immunoprecipitates were washed three times with PBS, and then analyzed via SDS-PAGE. The protein bands were visualized using ECL-Plus Western blot detection kit (Amersham BioSciences, UK).

### RNA-sequencing analysis

RNA-sequencing analysis (Aksomics, China) was carried out as per the user guide. HA-VSMCs were transfected with pcDNA3.1-SNHG1, along with its negative control (Con). Total RNA was extracted after 48-h transfection, and then mixed with oligo dT magnetic beads to enrich for mRNAs. The purified mRNAs were disrupted into short fragments and cDNAs were synthesized. After cDNAs library generation, sequencing was performed using the Illumina Hiseq™ 4000 platform (Illumina, Inc., USA). Differentially expressed genes (DEGs) were screened out by the limma package in R software (Lu et al., [12] based on the comparison of expression values between Control and SNHG1-overexpressed HA-VSMCs. The screen criteria for DEGs were as follows: |log_2_FC|> 2 and *P* < 0.05. Gene Ontology (GO) analysis was performed to reveal the functions and pathways of DEGs using the clusterProfiler package (Yu et al., [13]. A *P*-value < 0.05 was considered statistically significant. Volcano plots and bubble charts were drawn by using the ggplot2 package in R.

### *RNA fluorescence *in situ* hybridization*

Cy3-labeled SNHG1 and fluorescein isothiocyanate (FITC)-labeled Bhlhe40 mRNA probes were obtained from RiboBio (China). RNA fluorescence in situ hybridization (RNA-FISH) assays were performed using fluorescent in situ hybridization kit (RiboBio, China) following the protocol. In brief, cells were fixed in 4% formaldehyde, they were permeabilized in PBS containing 0.5% Triton X-100, and then they were pre-hybridizated in pre-hybridization solution. Next, probes were added in the hybridization solution and incubated with the cells overnight in the dark. Nuclei were counter-stained with 4′,6-diamidino-2-phenylindole (DAPI, Beyotime, China). Images were taken using a fluorescent microscope (Leica, Germany).

### RNA pull-down assay and RNA immunoprecipitation assay

RNA pull-down assays were performed using Pierce™ Magnetic RNA–Protein Pull-Down Kit (Thermo Fisher Scientific, USA). Briefly, biotin-labeled RNAs (SNHG1 and control) were incubated with HA-VSMCs cell lysates. Then, the complexes were isolated using Streptavidin Magnetic Beads. Lastly, the RNA and protein enriched in the complexes were purified and detected by qRT-PCR or Western blot, respectively. RNA immunoprecipitation (RIP) assays were performed using EZ-Magna RIP Kit (Millipore, USA) and following the manufacturer’s protocol.

### RNA stability assay

At 24 h after transfection, actinomycin D (ActD, 5 μg/ml) was added into media of transfected HA-VSMCs. At the indicated time points (0, 2, 4, 6, 8, 12 h) post ActD addition, RNA was extracted and Bhlhe40 mRNA stability was examined by qRT-PCR relative to time 0 and normalized to 18S rRNA.

### Immunofluorescence

Cells were grown on sterile glass coverslips, fixed in 4% paraformaldehyde for 15 min. After rinsing with PBS tree times, the cells were incubated with 0.5% Triton X-100 for 1 h and then blocked with bovine serum albumin for 30 min. Then samples were incubated with rabbit anti-Bhlhe40 (1:500, Proteintech, USA) at 4℃ overnight. After washing with PBS, the fluorescein-488 antibody (1:500, Invitrogen, USA) was added and the samples were incubated at room temperature for 1 h. Nuclei were labeled by DAPI (Beyotime, China). Immunofluorescence was analyzed using a fluorescent microscope (Leica, Germany).

### In vitro* SUMOylation assay*

HA-VSMCs were transfected with pcDNA3.1-SNHG1 (SNHG1) or its negative control (Con) and were lysed in lysis buffer containing 20 mM N-ethylmaleimide. After clearance by centrifugation, proteins were dissociated by being heated for 10 min. Samples were diluted with lysis buffer, and GFP-tagged Bhlhe40 was immunoprecipitated overnight at 4℃ with anti-GFP. Immunoprecipitated proteins were washed with lysis buffer and were analyzed by immunoblot with His-SUMO1 and Flag-PIAS3.

### Luciferase reporter assay

Luciferase reporter plasmids of wild-type Atg10 or E-box deletion mutants were used in the experiments. HA-VSMCs were seeded in 96-well culture plates and cotransfected with luciferase reporter plasmids and adenovirus vector expressing Bhlhe40 or empty vector, respectively. After 48 h, luciferase activity in cells was measured using the Dual-Luciferase Reporter Assay System (Promega, USA). Firefly activity was normalized to renilla luciferase activity.

### Chromatin immunoprecipitation assay

This procedure was performed as previously described [11]. In brief, anti-Bhlhe40 and control anti-IgG were used to immunoprecipitate the relevant protein-DNA complex. Then, the precipitated DNAs were detected by qRT-PCR. The primer sequences are shown in Supplementary Table 3.

### Tandem mRFP-GFP fluorescence microscopy

To monitor the autophagic flux, HA-VSMCs were transfected with tandem fluorescent mRFP-GFP-LC3 adenovirus for 48 h, and then cells were treated with indicated treatment for another 12 h. Images of samples were acquired using confocal fluorescence microscopy. Yellow (overlay of GFP and mRFP) fluorescence spots represented early autophagosomes, red (mRFP alone) fluorescence spots indicated late autophagosomes.

### Statistical analysis

Data were analyzed using SPSS 19.0 software (SPSS, USA). Values were presented as mean ± standard deviation (SD). A student’s *t* test or one-way analysis of variance was used to analyze significant differences between the outcomes of two groups or multiple groups, respectively. Statistical significance was established at *P* < 0.05.

## Results

### LncRNA SNHG1 is identified and its low expression is correlated with HG-induced calcification/senescence of HA-VSMCs

**Fig. 1:**
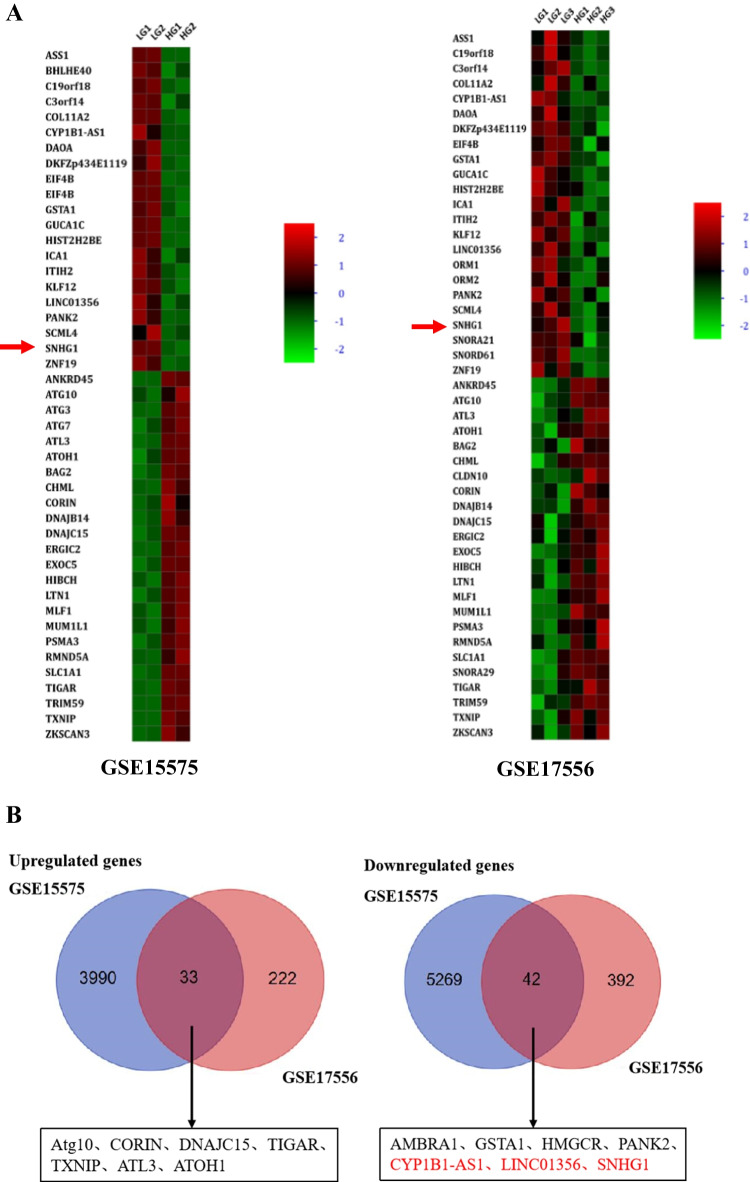

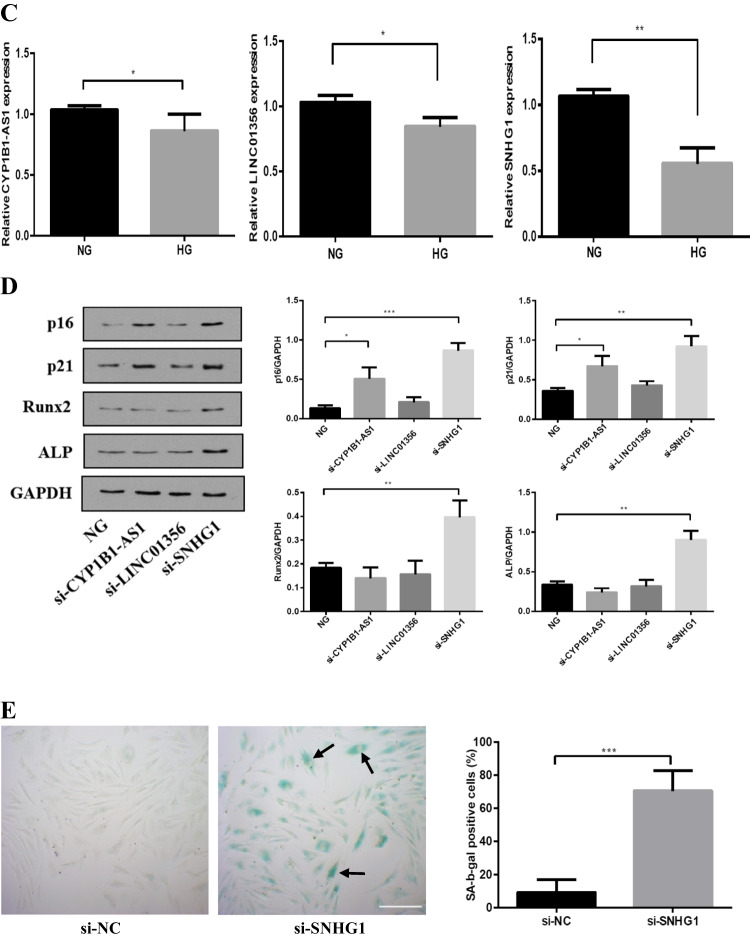

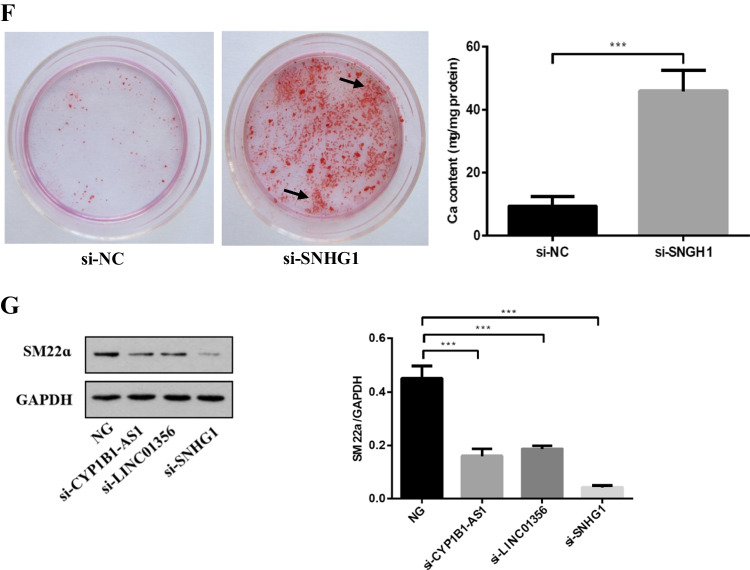
LncRNA SNHG1 is downregulated in HG-induced HA-VSMCs. **A** Microarray heatmaps of DEGs between HG-induced cells from NG-induced cells. The color “green” and “red” denote low and high expression, respectively. **B** Venn diagrams of the overlapping DEGs. Three lncRNAs were downregulated in HG-induced HA-VSMCs as compared to that in NG group. **C** Relative expression levels of lncRNAs SNHG1, CYP1B1-AS1 and LINC01356 in HG and NG groups were quantified by qRT-PCR. **D–G** The effects of these three lncRNAs on HA-VSMCs calcification/senescence and contractile phenotype. HA-VSMCs were treated with NG, NG with si-CYP1B1-AS1, NG with si-LINC01356, and NG with si-SNHG1, respectively. **D** The protein levels of p16, p21, Runx2, and ALP were determined by Western blot in the above four groups; GAPDH was loading control. Representative images of (**E**) SA-β-gal staining and (**F**) Alizarin Red S staining in HA-VSMCs under conditions of NG with si-NC or NG with si-SNHG1. Calcium content was extracted with cetylpyridinium chloride and quantified by spectrophotometry; the red area indicated by the arrow is the calcium accumulation. Semiquantitative analysis of SA-β-gal-positive cells was performed using Image J (200 × magnification); the blue area indicated by the arrow is the positive staining of SA-β-gal, scale bar = 100 µm. G The protein level of SM22ɑ was determined by Western blot in the above four groups; GAPDH was loading control. Results shown are means ± SD from triplicate experiments. ^*^, *P* < 0.05; ^**^, *P* < 0.01; ^***^, *P* < 0.001

To explore aberrantly expressed lncRNAs in HG condition, we analyzed the lncRNA expression profiles between NG- and HG-induced HA-VSMCs/HEK293 cells based on the GSE datasets (GSE17556 and GSE15575). Analysis of the GSE17556 dataset led to the identification of 689 genes with 255 genes being upregulated and 434 genes being downregulated in HG group compared with NG group. Analysis of the GSE15575 dataset led to the identification of 9334 genes with 4023 genes being upregulated and 5311 genes being downregulated in HG group compared with NG group. Then, we used Venn diagram software to identify the commonly DEGs in the two datasets. Results showed that a total of 75 commonly DEGs (33 upregulated and 42 downregulated genes) were detected, including 3 downregulated lncRNAs (SNHG1, CYP1B1-AS1, LINC01356) in the HG groups (Fig. 1A, B). Meanwhile, qRT-PCR results revealed that lncRNA SNHG1 was the most downregulated lncRNA of the 3 lncRNAs (Fig. 1C).

Next, we explored the biological functions of these 3 lncRNAs in HG-induced calcification/senescence of HA-VSMCs, and we established the loss-of-function cell models by trasfection a specific siRNA against CYP1B1-AS1, LINC01356, and SNHG1 into HA-VSMCs. LncRNAs expression levels were significantly reduced after siRNA transfection in HA-VSMCs (Fig. S1A-C). The results showed that knockdown of SNHG1 under NG condition significantly increased the levels of calcification markers ALP and Runx2, and senescence markers p16 and p21 proteins, as well as staining of SA-β-gal and Alizarin Red S-positive cells (Fig. 1D–F). Furthermore, knockdown of SNHG1 under NG condition decreased the expression of the VSMCs contractile marker SM22ɑ (Fig. 1G). Thus, we focused on SNHG1 for further studies.

### SNHG1 positively regulates Bhlhe40 mRNA and protein expression levels

**Fig. 2:**
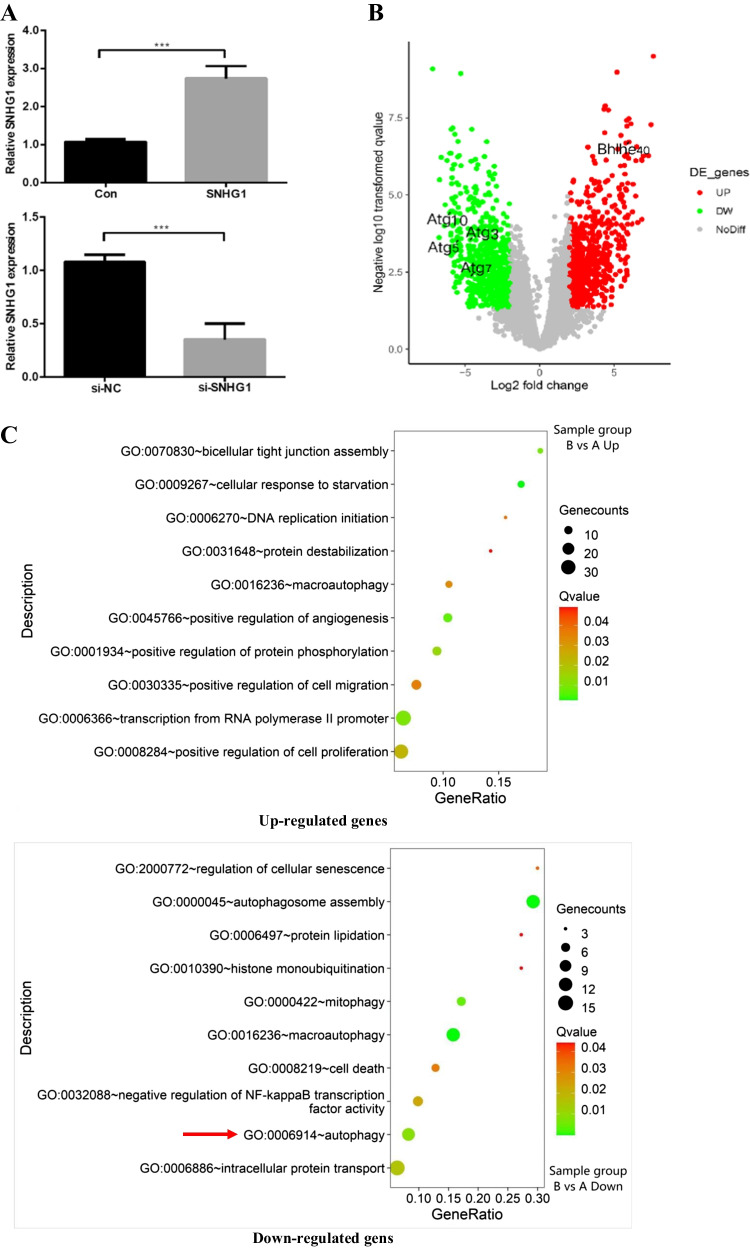

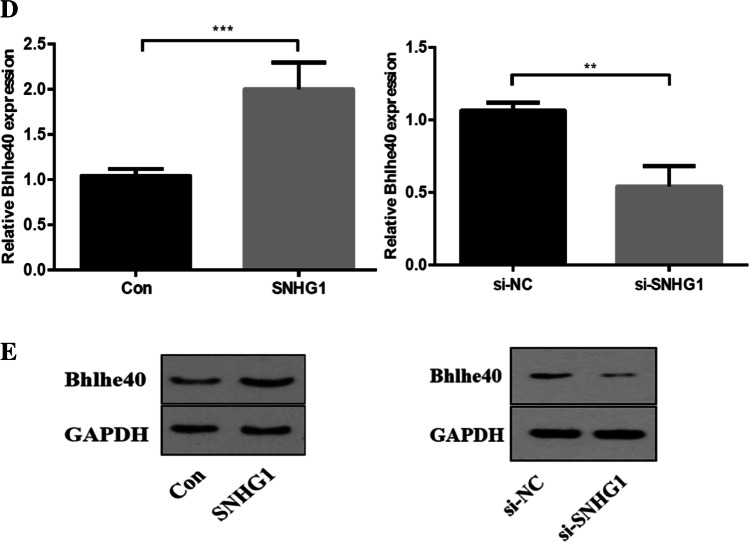
SNHG1 positively regulates Bhlhe40 expression in HA-VSMCs. **A** The overexpression (SNHG1) and knockdown (si-SNHG1) efficiencies of SNHG1 were assessed by qRT-PCR. **B** Volcano plot of DEGs between SNHG1-overexpressed HA-VSMCs from control group. **C** GO analysis of the DEGs. **D** Bhlhe40 mRNA levels were evaluated in indicated transfectants using qRT-PCR. **E** Cell extracts underwent Western blot for determination of Bhlhe40 protein expression; GAPDH was loading control. Results shown are means ± SD from triplicate experiments. ^**^, *P* < 0.01; ^***^, *P* < 0.001

To further explore the effects of SNHG1 on HG-induced HA-VSMCs senescence/calcification, we constructed SNHG1-overexpressed or knockdown HA-VSMCs via transfecting SNHG1 overexpression plasmid or SNHG1 specific siRNA, respectively. The transfecting efficiency was verified using qRT-PCR (Fig. 2A). Then, we examined the mRNA expression profiles in SNHG1-overexpressed HA-VSMCs. RNA-seq results identified a total of 1650 DEGs when comparing the SNHG1-overexpressed HA-VSMCs with the negative control cells, which included 920 upregulated and 730 downregulated DEGs with |log_2_FC|> 2. The volcano plot is shown in Fig. 2B. Among the list of DEGs, we noted that a transcription factor Bhlhe40, was significantly increased in SNHG1 overexpression group compared with that in the control group with transfecting empty plasmid (Fig. 2B). The results, through GO analysis, indicated that upregulated genes were mainly involved in bicellular tight junction assembly, while downregulated genes mainly enriched in autophagy signaling pathway (Fig. 2C), which has been proven to be closely related to cellular senescence [14].

As displayed in Fig. 2D and E, the expression of Bhlhe40 was increased in SNHG1 group and decreased in si-SNHG1 group when compared with their negative controls, at both mRNA and protein levels.

### *SNHG1 stabilizes Bhlhe40 mRNA *via* RNA-RNA interaction*

**Fig. 3:**
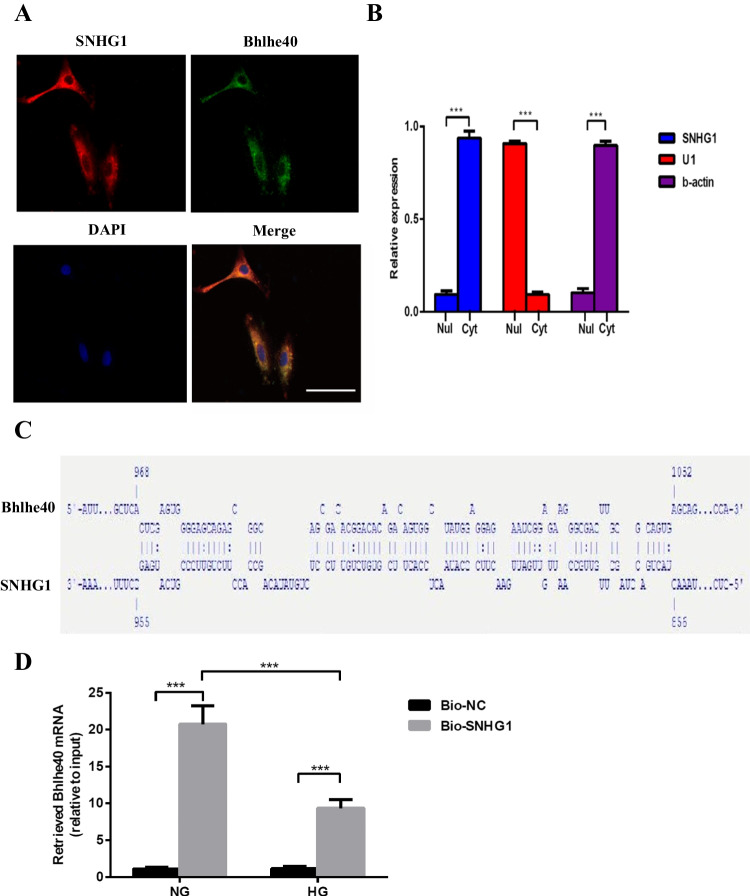

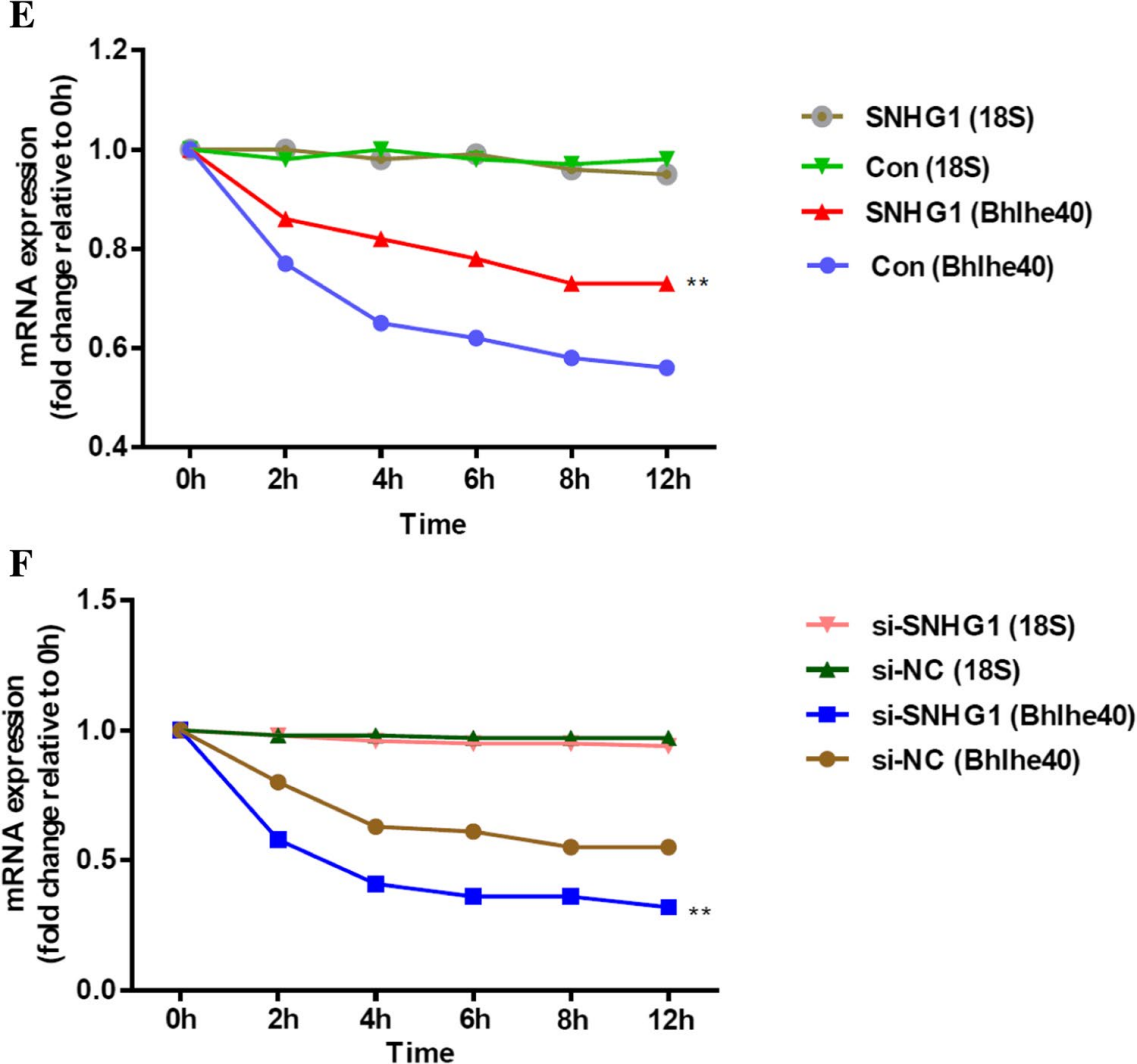
SNHG1 stabilizes Bhlhe40 mRNA via RNA-RNA interaction. **A** Subcellular localization of SNHG1 was detected by RNA-FISH assay in HA-VSMCs; SNHG1 probes are red; Bhlhe40 probes are green (200 × magnification), scale bar = 100 µm. **B** Nuclear and cytoplasmic fractions of HA-VSMCs were subjected to qRT-PCR. U1 is the nuclear (Nul) positive control; β-actin is the cytoplasmic (Cyt) positive control. **C** Schematic diagram of the predicted interaction sequences between SNHG1 and Bhlhe40 mRNA by IntaRNA. **D** The RNA-RNA interaction between Bhlhe40 mRNA and SNHG1 was detected by RNA pull-down assay using in vitro transcribed biotin-labeled SNHG1. The retrieved RNA was quantified by qRT-PCR and displayed as percentage of input RNA. **E**, **F** After transfecting SNHG1 overexpression plasmid (**E**) or specific si-SNHG1 (**F**) into HA-VSMCs, the stability of Bhlhe40 mRNA over time was measured by qRT-PCR relative to time 0 after blocking new RNA synthesis with ActD (5 μg/ml) and normalized to 18S rRNA. Results shown are means ± SD from triplicate experiments. ^**^, *P* < 0.01; ^***^, *P* < 0.001

Due to the significant correlation between SNHG1 and Bhlhe40 in HA-VSMCs, we next investigated how SNHG1 promotes Bhlhe40 expression. We first performed RNA-FISH and RNA fractionation of the nucleus and cytoplasm to examine the subcellular localization of SNHG1. The data revealed that SNHG1 was mainly located in the cytoplasm of HA-VSMCs (Fig. 3A, B). We further dissected the subcellular localization of Bhlhe40 mRNA and found that Bhlhe40 mRNA was co-localized with SNHG1 in the cytoplasm of HA-VSMCs (Fig. 3A). Cytoplasmic lncRNAs have been shown to regulate the stability and expression of target mRNAs via RNA-RNA interaction [15, 16]. Therefore, we explored whether SNHG1 and Bhlhe40 mRNA had interaction region using IntaRNA (http://rna.informatik.uni-freiburg.de/IntaRNA/Input.jsp). Intriguingly, we predicted a long interaction region between SNHG1 and 3′UTR of Bhlhe40 mRNA with the binding sites at 857–954 nucleotides of SNHG1 and at 969–1051 nucleotides of Bhlhe40 mRNA (Fig. 3C). The physical interaction between SNHG1 and Bhlhe40-3′UTR was further validated by RNA pull-down assay. The pull down of biotin-labeled SNHG1 in HA-VSMCs was significantly enriched for Bhlhe40 mRNA compared to the negative control (Fig. 3D). To further investigate whether the RNA-RNA interaction between SNHG1 and Bhlhe40 mRNA could enhance the stability of Bhlhe40 mRNA, we used ActD (5 μg/ml), an inhibitor of RNA polymerase II [16], to block new RNA synthesis in HA-VSMCs. Then, Bhlhe40 mRNA levels were measured and were referenced to 18S-RNA, which is not affected by nuclease. As shown in Fig. 3E, overexpression of SNHG1 in HA-VSMCs increased the stability of Bhlhe40 mRNA compared with HA-VSMCs transfected with an empty vector. In contrast, the stability of Bhlhe40 mRNA was decreased in HA-VSMCs transfected with si-SNHG1 relative to the control (Fig. 3F). Take together, these results suggested that SNHG1 directly binds to the Bhlhe40-3′UTR and stabilizes Bhlhe40 mRNA.

### SNHG1 promotes Bhlhe40 protein nuclear translocation by enhancing the SUMOylation of Bhlhe40 at K279 residue

**Fig. 4:**
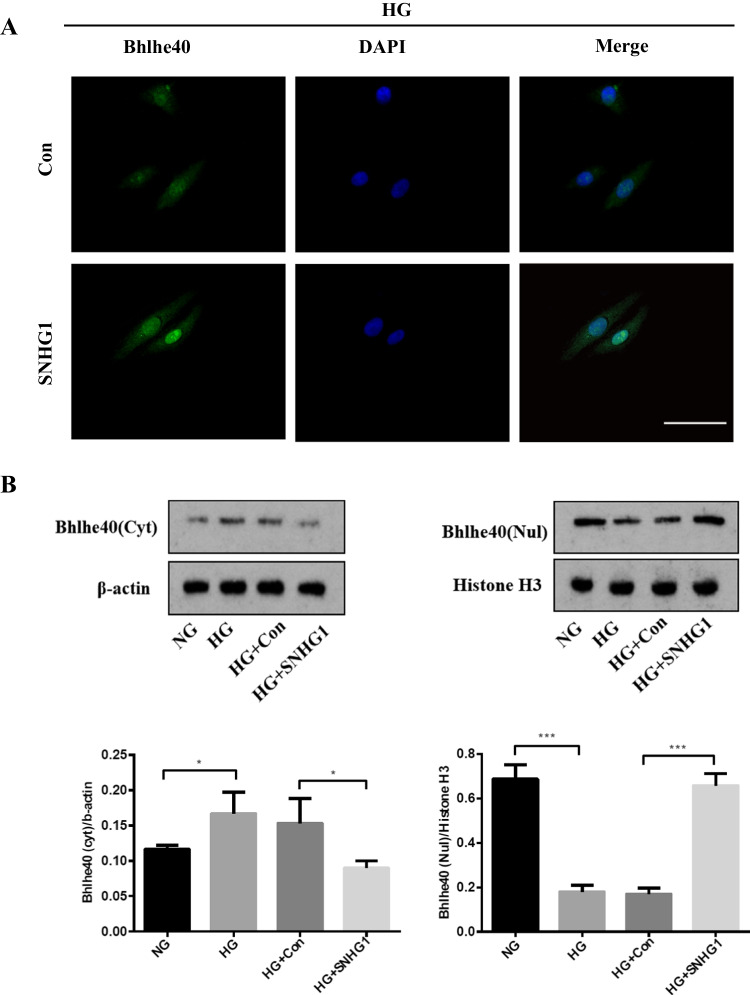

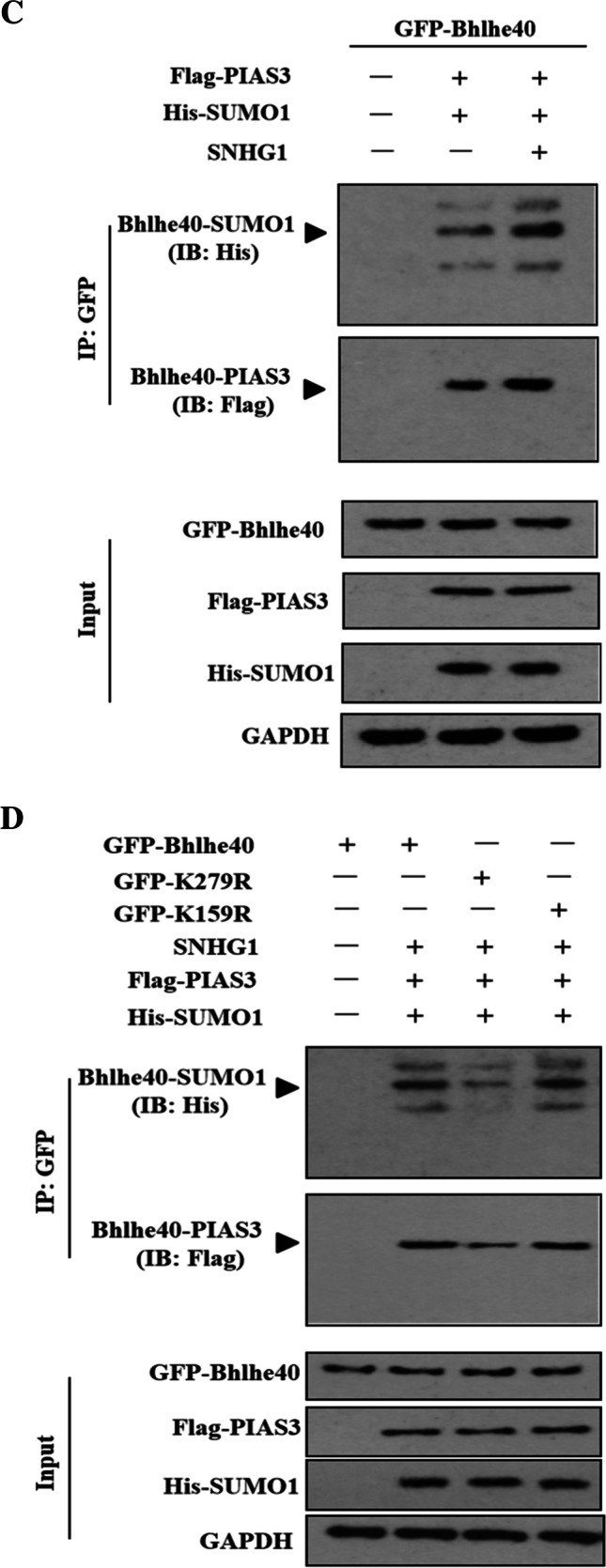

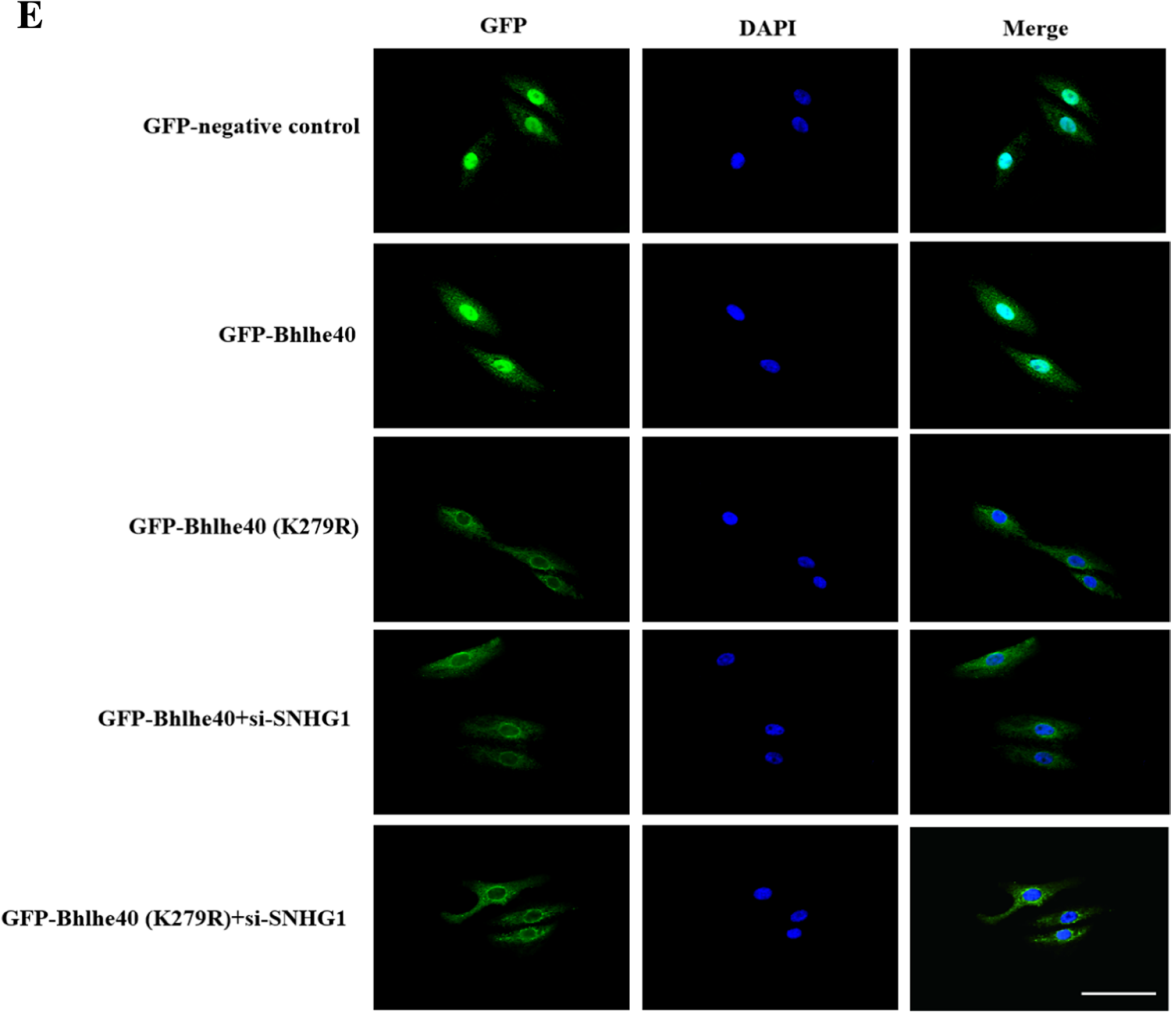

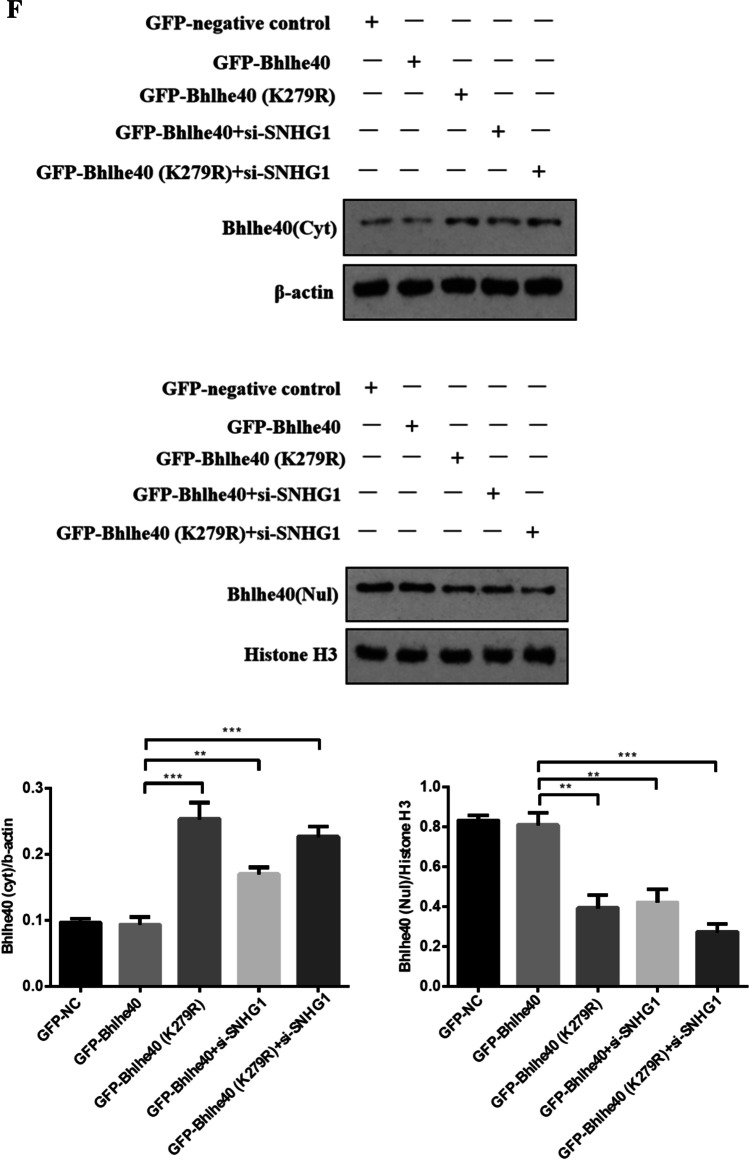
SNHG1 effectively promotes Bhlhe40 protein nuclear translocation by enhancing the SUMOylation of Bhlhe40 at K279. **A** Immunofluorescence assay of the localization of Bhlhe40 protein (green) in HA-VSMCs transfected with empty vector (Con) or pcDNA3.1-SNHG1 (SNHG1, 200 × magnification), scale bar = 100 µm. **B** The expression of Bhlhe40 protein in nucleus (Nul) and cytoplasm (Cyt) of SNHG1-overexpressing HA-VSMCs were shown by Western blot. Nuclear segregation is assayed by Histone H3; cytoplasmic segregation is assayed by β-actin. **C** Immunoblot analysis was performed to detect the modification of Bhlhe40 by SUMO1 after SNHG1 overexpression. Cells lysates were subjected to immunoprecipitation by anti-GFP followed by immunoblot with anti-Flag or anti-His. **D** Immunoblot analysis of the SUMOylation of Bhlhe40 in HA-VSMCs transfected with Bhlhe40-WT or point mutants (K159R, K279R). Cells lysates were subjected to immunoprecipitation by anti-GFP followed by immunoblot with anti-Flag or anti-His. **E** Immunofluorescence assay was used to detect the location of Bhlhe40 protein in HA-VSMCs (200 × magnification), scale bar = 100 µm. The cells were transfected with pcDNA3.1-SNHG1, Bhlhe40-WT or point mutants (K159R, K279R). **F** The expression of Bhlhe40 protein in nucleus (Nul) and cytoplasm (Cyt) of HA-VSMCs were detected by Western blot. The cells were transfected with pcDNA3.1-SNHG1, Bhlhe40-WT or point mutants (K159R, K279R). Nuclear segregation is assayed by Histone H3; cytoplasmic segregation is assayed by β-actin

As a transcription factor, Bhlhe40 protein translocation to the nucleus is a necessary step for its transcriptional activity (Kiss, Mudryj, & Ghosh [17]. Therefore, we wondered whether SNHG1 affects Bhlhe40 protein nuclear translocation. We used immunofluorescence and subcellular fractionation to detect the subcellular distributions of Bhlhe40 protein in control and SNHG1-overexpressed HA-VSMCs. The results showed that Bhlhe40 protein was mainly located in the cytosol in control group (Fig. 4A, B). Notably, such a distribution pattern of Bhlhe40 was found being regulated by SNHG1. Overexpression of SNHG1 in HA-VSMCs led to the increasement of Bhlhe40 protein in the nucleus with reducing its level in the cytoplasm (Fig. 4A). Western blot data also showed that overexpression of SNHG1 increased the protein level of Bhlhe40 in the nuclear fraction (Fig. 4B). These findings indicated that SNHG1 induced Bhlhe40 protein localization into the nucleus.

Mechanistically, it is still unclear how SNHG1 promoted the nuclear translocation of Bhlhe40 protein. SUMOylation is a post-translational modification and is known to affect the nuclear translocation of targeted proteins [7]. It has been previously reported that Bhlhe40 protein could be SUMOylated by SUMO1 in HEK293 cells [18]. Indeed, we found that SNHG1 enhanced the SUMOylation modification of SUMO1 on Bhlhe40 (Fig. 4C). These results suggested that SNHG1 may promote Bhlhe40 protein nuclear translocation by enhancing Bhlhe40 SUMOylation. Previous studies have shown that the lysine at 159 (K159) and 279 (K279) served as the major SUMOylation sites of Bhlhe40 [18]. We then mutated these lysines to arginines at each site individually (K159R mutant and K279R mutant respectively). We found that both Bhlhe40 and K159R mutant were SUMOylated. In contrast, SUMOylated Bhlhe40 was decreased markedly in K279R mutant, suggesting that K279 is the residue that covalently binds SUMO (Fig. 4D). Finally, as shown in Fig. 4E and F, whereas wild-type Bhlhe40 was mainly located in the nucleus of HA-VSMCs, knockdown of SNHG1 or K279R mutant of Bhlhe40 markedly impaired nuclear localization of Bhlhe40. The above results indicated that Bhlhe40 SUMOylation at K279 is necessary for SNHG1- induced Bhlhe40 nuclear translocation.

### SNHG1 enhances Bhlhe40 SUMOylation by acting as a scaffold to promote the binding of Bhlhe40 and PIAS3

**Fig. 5:**
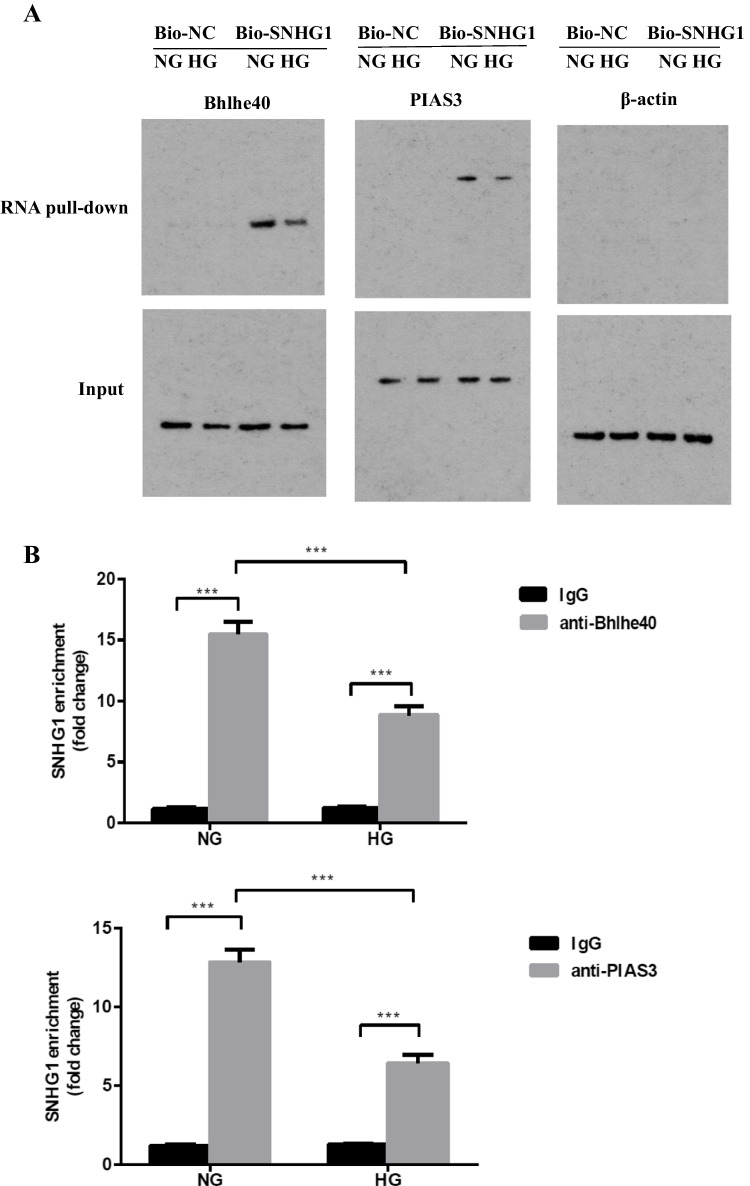

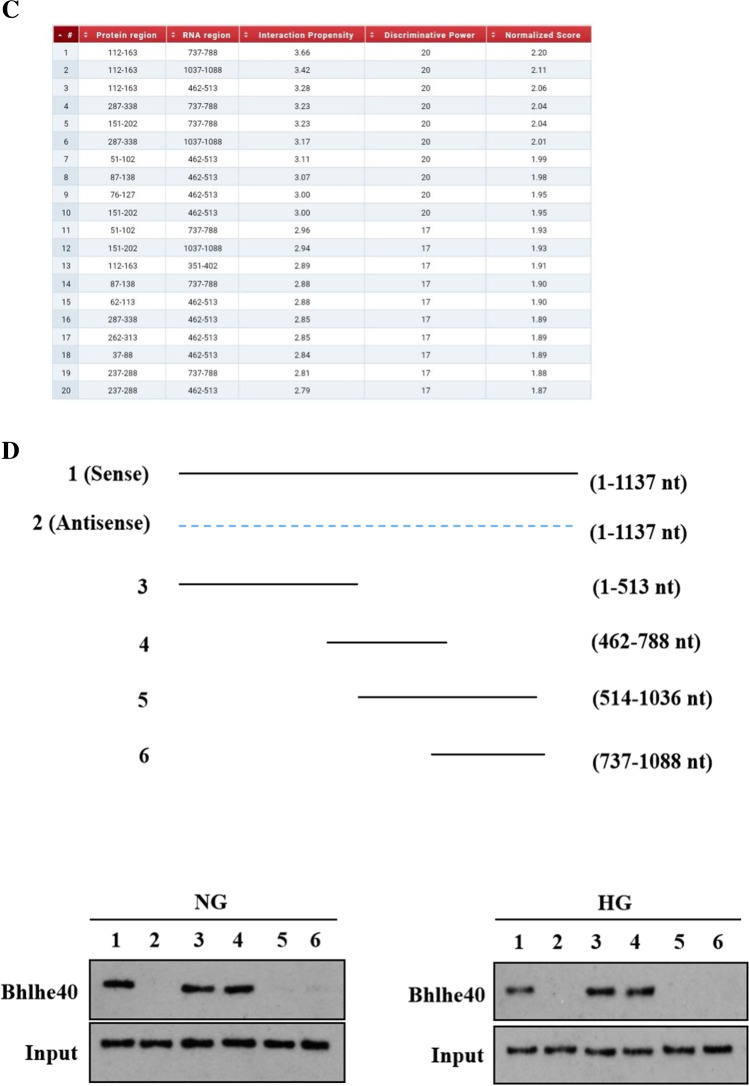
SNHG1 functions as a scaffold for Bhlhe40/PIAS3 to regulate Bhlhe40 SUMOylation. **A** Western blot analysis of the proteins retrieved from the SNHG1 pull-down assay using anti-Bhlhe40 and anti-PIAS3. **B** RIP assays were performed using anti-Bhlhe40 or anti-PIAS3 antibodies in HA-VSMCs. qRT-PCR was used to measure the enrichment of SNHG1. **C** Online-predicted binding sites between SNHG1 and Bhlhe40 protein on CatRAPID database. **D** RNA pull-down assays were used to examine the interaction between Bhlhe40 and the different mutants of SNHG1. Results shown are means ± SD from triplicate experiments

We next investigated how SNHG1 enhances Bhlhe40 protein SUMOylation. LncRNAs may function as a scaffold to facilitate the interacting between transcriptional factor and E3 ubiquitin ligase or protein, and regulate the degradation or localization of transcriptional factor [19, 20]. Given that PIAS3, a SUMO E3 ligase, could enhance the SUMOylation of Bhlhe40 protein [18], we hypothesized that SNHG1 may enhance Bhlhe40 SUMOylation by serving as a scaffold to bring Bhlhe40 and PIAS3 together. As expected, our results revealed that biotin-labeled SNHG1, but not the control fragments, specifically retrieved Bhlhe40 and PIAS3 (Fig. 5A). Consistently, RIP assays showed enrichment of SNHG1 in complexes precipitated by anti-Bhlhe40 or anti-PIAS3 as compared with control IgG (Fig. 5B).

To identify which region of SNHG1 binds to Bhlhe40 protein, we constructed different lengths of SNHG1 mutants based on the predicted binding sites of SNHG1 in the CatRAPID database (http://s.tartaglialab.com/page/catrapid_group) (Fig. 5C), and then performed RNA pull-down assays with Bhlhe40 antibody. We found that 462–513 nt of SNHG1 is important for the binding between SNHG1 and Bhlhe40 (Fig. 5D). Collectively, these results indicated that SNHG1 facilitated Bhlhe40 SUMOylation by acting as a scaffold for Bhlhe40 and PIAS3.

### SNHG1 protects HA-VSMCs from HG-induced calcification/senescence by suppression of Atg10 and autophagy

**Fig. 6:**
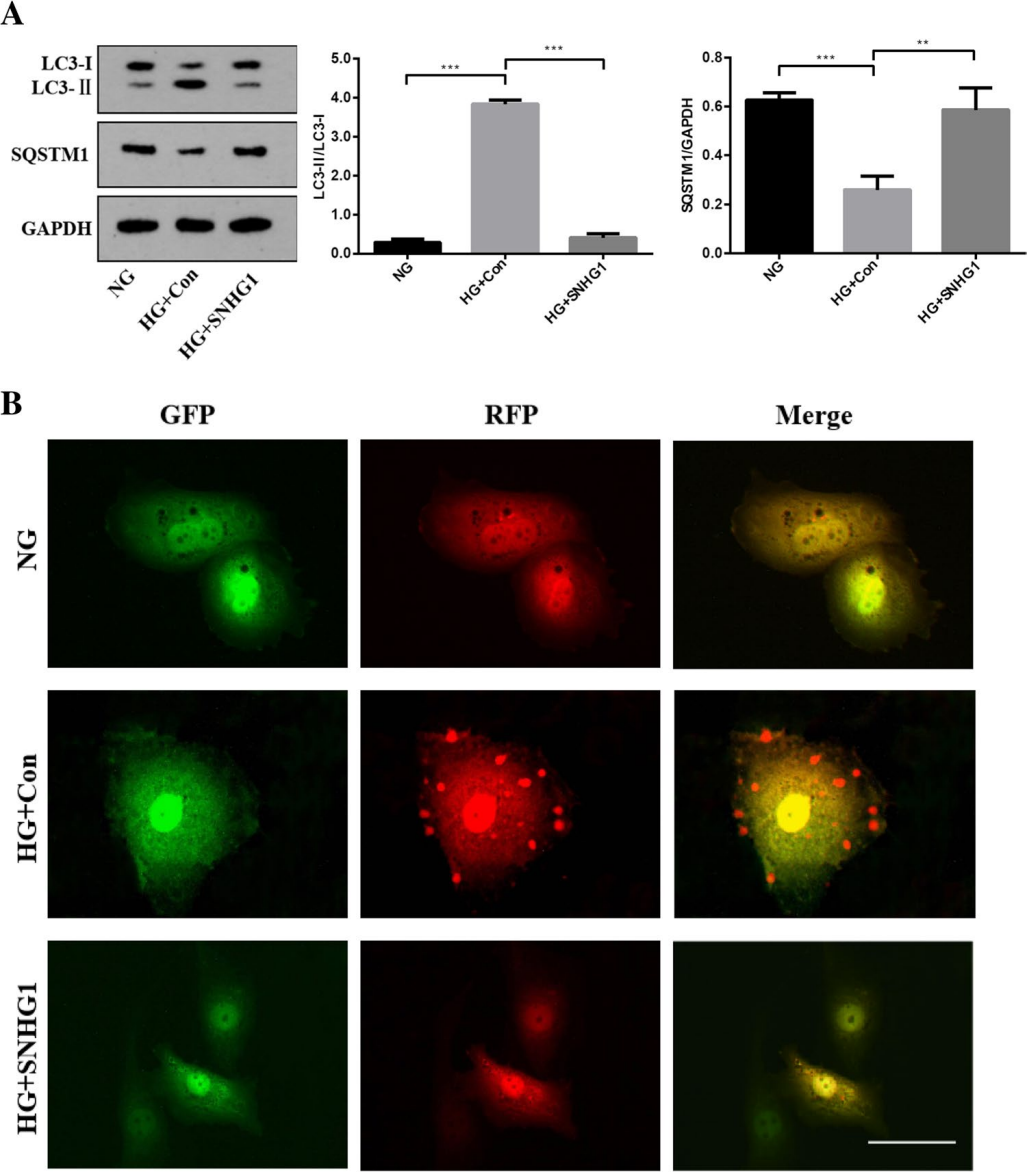

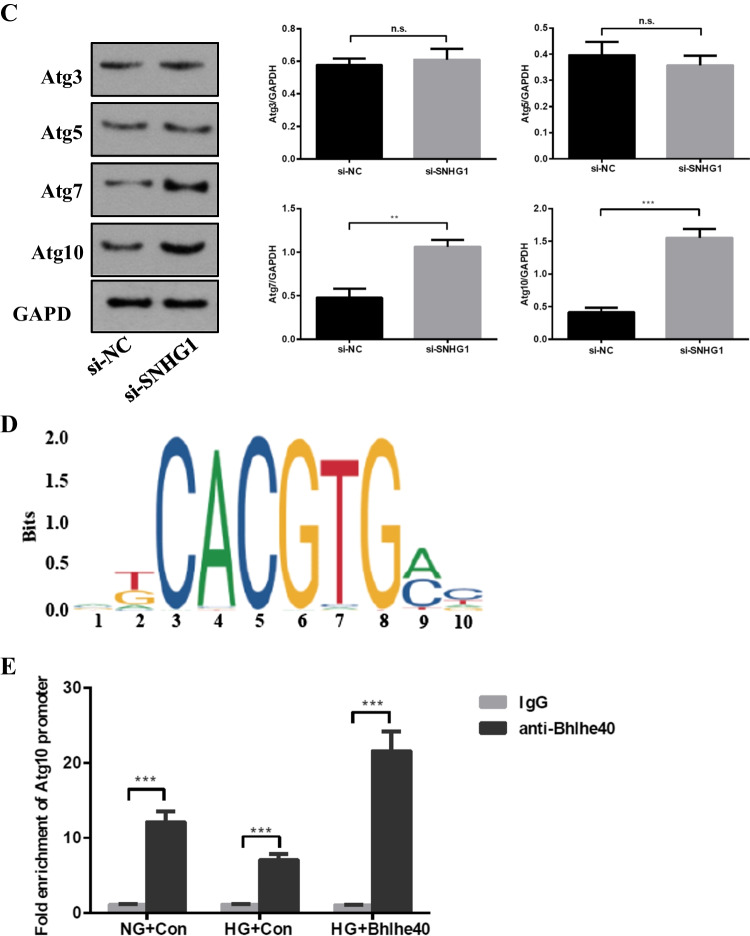

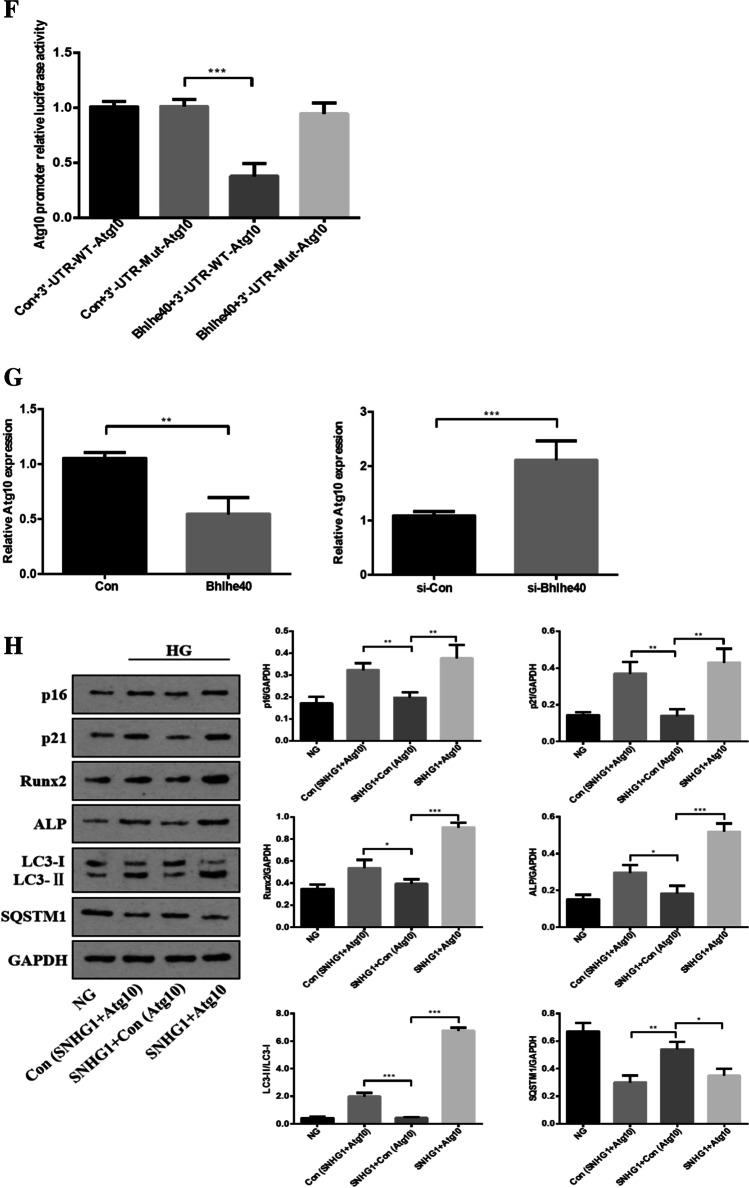

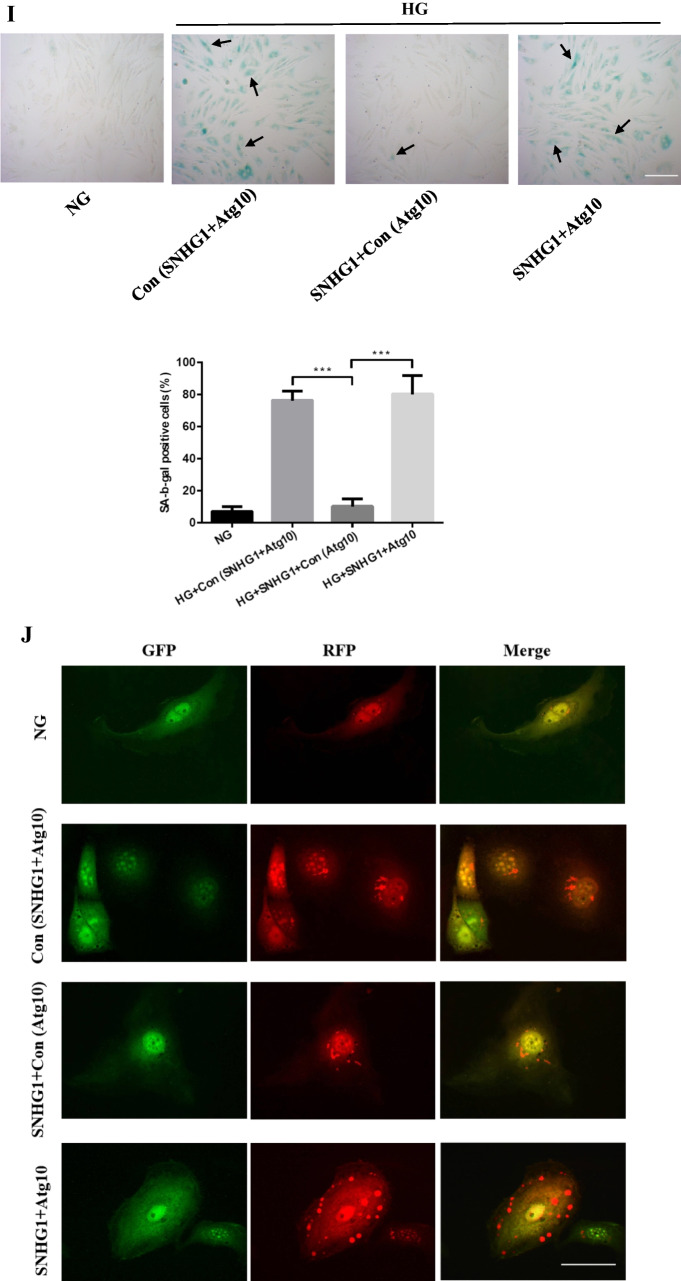
SNHG1 regulates Bhlhe40 mediated Atg10 transcription inhibition. **A** Western blot was performed to detect the levels of autophagy markers in HA-VSMCs after transfection with pcDNA3.1-SNHG1. **B** mRFP-GFP-LC3 distribution in HA-VSMCs transfected with mRFP-GFP-LC3 and pcDNA3.1-SNHG1 were analyzed by confocal microscopy (200 × magnification), scale bar = 100 µm. **C** The protein levels of Atg3, Atg5, Atg7 and Atg10 were measured in HA-VSMCs after transfection of si-SNHG1 by Western blot. **D** The binding sites of Bhlhe40 on Atg10 promoter region and the binding sequence was obtained using the JASPAR website. **E** ChIP assays were performed to verify the reliability of the binding sequence of Bhlhe40 on Atg10 promoter region. **F** HA-VSMCs were transfected with luciferase reporter carrying WT-pGL3-Atg10 or Mut-pGL3-Atg10 and cotransfected with the adenovirus vector expressing Bhlhe40 (Bhlhe40) or not (Con). Firefly luciferase values, normalized for Renilla luciferase, are presented. **G** qRT-PCR analysis of Atg10 in HA-VSMCs transfected with the adenovirus vector expressing Bhlhe40 or si-Bhlhe40. **H** HA-VSMCs were transfected with Atg10 overexpression plasmid (Atg10) or negative control (Con), respectively. Then, p16, p21, Runx2, ALP, LC3-II, and SQSTM1 protein levels were measured. **I** Representative images of SA-β-gal staining in the above four groups; semiquantitative analysis of SA-β-gal-positive cells was performed using Image J (200 × magnification); the blue area indicated by the arrow is the positive staining of SA-β-gal, scale bar = 100 µm. **J** mRFP-GFP-LC3 distribution in HA-VSMCs transfected with mRFP-GFP-LC3, pcDNA3.1-SNHG1 and Atg10 overexpression plasmid were analyzed by confocal microscopy (200 × magnification), scale bar = 100 µm. Results shown are means ± SD from triplicate experiments

To explore the common biological processes that are affected by SNHG1, we performed GO analysis on the DEGs. Strikingly, GO analysis revealed suppression of autophagy pathway as a result of SNHG1 overexpression (Fig. 2C). Measurement of the conversion of LC3-I to LC3-**II**, together with the sequestosome 1 (SQSTM1) level, is regarded to be well correlated with autophagic flux [21]. Indeed, we found that HG increased LC3-II/LC3-I levels and reduced SQSTM1 expression in HA-VSMCs, while overexpression of SNHG1 reduced LC3-II/LC3-I levels and increased SQSTM1 expression (Fig. 6A). Meanwhile, overexpression of SNHG1 inhibited autophagy, as shown by the decreases in mRFP-GFP-LC3 puncta accumulation (Fig. 6B). These results suggested that SNHG1 is involved in autophagy of HA-VSMCs. Further, the expression of DEGs involved in autophagy pathway, including Atg3, Atg5, Atg7, and Atg10, were tested by Western blot analysis. As shown in Fig. 6C, all of these four filtered DEGs showed a higher expression in SNHG1-knockdown group in comparison with the control group. Among these, Atg10 was chosen for further research, for its highest fold change.

Next, we asked how SNHG1 regulates Atg10 expression. It is well known that when Bhlhe40 translocates into the nucleus, it binds to target gene promoters containing the E-box hexanucleotide sequence (CANNTG), and then represses transcription (Kiss, Mudryj, & Ghosh [17]. Further we wanted to know whether SNHG1 regulated Atg10 expression via Bhlhe40. We found that Atg10 contains a typical E-box in the promoter using the JASPAR database (Fig. 6D). This suggests that it exists a regulatory mechanism between Bhlhe40 and Atg10. The subsequent chromatin immunoprecipitation (ChIP) assay supported the notion that Bhlhe40 could directly bind to the E-box in Atg10 promoter (Fig. 6E). Furthermore, the luciferase reporter assay showed that overexpression of Bhlhe40 significantly decreased the relative luciferase activity of the WT-Atg10 reporter, whereas Bhlhe40 had no effect on luciferase activity of Mut-Atg10 reporter, in which the putative Bhlhe40 binding site in Atg10 was mutated (Fig. 6F). Consistent with this repression, overexpression of Bhlhe40 decreased the Atg10 mRNA level in HA-VSMCs, while knockdown of Bhlhe40 had completely opposite effect (Fig. 6G).

We then assessed the ability of Atg10 to abrogate the SNHG1-mediated alleviation of HG-induced HA-VSMCs calcification/senescence. As expected, overexpression of Atg10 abrogated the SNHG1-mediated repression of ALP, Runx2, p16, and p21 (Fig. 6H), same as the SA-β-gal staining (Fig. 6I). We further found that SNHG1-suppressed autophagy could be reversed by Atg10 overexpression (Fig. 6H, J). Collectively, these findings indicated that Atg10 is the target of Bhlhe40 and SNHG1 could perform its functions partly depends on regulation of Bhlhe40 mRNA stability and protein nuclear translocation.

## Discussion

The major findings of the present study were as follows: (1) SNHG1 reduction is related to the HG-induced HA-VSMCs calcification/senescence. (2) In HG condition, overexpression of SNHG1 ameliorates the calcification/senescence of HA-VSMCs by suppressing Atg10 expression and over-activated autophagy. (3) SNHG1 directly interacts with Bhlhe40 mRNA 3′UTR, increases Bhlhe40 mRNA stability, and upregulates Bhlhe40 mRNA expression level via RNA-RNA interaction. (4) SNHG1 is an enhancer of Bhlhe40 protein SUMOylation at K279, which in turn facilitates the nuclear translocation of Bhlhe40 protein. Bhlhe40 protein located in the nuclear results in transcriptional repression of Atg10. To our knowledge, this is the first report that SNHG1 could regulate the stability of mRNA and SUMOylation of protein, and these discoveries also enrich the regulation mechanisms of SNHG1 for post-transcriptional modification.

Hyperglycaemia induced by diabetes has been reported to cause senescence/calcification of HA-VSMCs, which results in the loss of arterial function, vascular remodeling, and the development of diabetic macrovascular complications [4, 22]. Aberrantly expressed lncRNAs play vital roles in diabetic macrovascular complications due to their biological functions (Leung, Amaram, & Natarajan [23]. However, the detail roles and molecular mechanisms of lncRNAs in HG-induced HA-VSMCs calcification/senescence remain to be clarified. SNHG1 was initially identified as a cancer-related lncRNA by several studies [8, 9]. Recently, Ma et al. [24] reported that SNHG1 was significantly upregulated in serum samples of patients with restenosis,inhibition of SNHG1 served a protective role against restenosis by suppressing HA-VSMCs proliferation and migration. However, the effects of SNHG1 on the pathologic changes of blood vessels may be dependent on the different pathological states. In this study, SNHG1 was first proved to be the executor of HG-induced HA-VSMCs calcification/senescence. In HG condition, overexpression of SNHG1 inhibits the calcification/senescence of HA-VSMCs, implying that SNHG1 would be a potential therapeutic target for diabetic vascular calcification/aging.

A spate of reports show that lncRNAs can function as decoys, guides, or scaffolds to combine DNA, RNA, or protein and exert diverse biological functions [8, 20]. Hence, to explore the mechanisms of SNHG1 in regulating HG-induced HA-VSMCs calcification/senescence, we used bioinformatics prediction and searched for mRNAs that SNHG1 might combine with. We found that forced expression of SNHG1 in HA-VSMCs resulted in increased Bhlhe40 mRNA and protein expression levels. Bhlhe40 is a transcription factor, which belongs to the basic helix-loop-helix protein family (Kiss, Mudryj, & Ghosh [17]. Our previous studies and many other studies have proved that Bhlhe40 is essential for multiple cellular functions, including regulation of blood pressure [25], HA-VSMCs calcification/senescence [11], alveolar macrophage self-renewal (Rauschmeier et al., [23], and in regulation of unsaturated fatty acid and glucose oxidative metabolism [26]. The co-localization of SNHG1 with Bhlhe40 mRNA in the cytoplasm of HA-VSMCs promoted us to investigate post-transcriptional regulation of SNHG1. Recent studies have indicated that lncRNAs could regulate mRNA stability and/or expression via RNA-RNA interaction at post-transcriptional level [15], [27]. Intriguingly, our RNA stability assay provided evidence that SNHG1 and Bhlhe40 mRNA 3′UTR are capable of forming an RNA-RNA duplex. This duplex protects Bhlhe40 mRNA from RNase degradation, thereby increasing its stability and expression.

Bhlhe40 is a transcriptional repressor that binds to target gene promoters in the nucleus (Kiss, Mudryj, & Ghosh [17]. Therefore, it is necessary for Bhlhe40 protein to shuttle from the cytoplasm to the nucleus (Kiss, Mudryj, & Ghosh [17]. Our results showed that the nuclear Bhlhe40 protein is significantly reduced after HG treatment. Regulation roles of lncRNAs in the lncRNA-protein interaction involve various aspects, including altering protein localization [28]. However, whether SNHG1 is involved in regulating the nuclear translocation of Bhlhe40 protein in HA-VSMCs remains largely unknown. Here, we presented proof of principle that SNHG1 overexpression facilitated the nuclear translocation of Bhlhe40 protein. SUMOylation is a post-translational modification similar to protein ubiquitinylation which primarily influences the stability and subcellular localization of target proteins [29],Walters et al., [12]. Wang et al. identified that Bhlhe40 could be modified by SUMO1 at K159 and K279, whereas PIAS3, as a SUMO E3 ligase, enhances the SUMOylation of Bhlhe40 [18]. Kunz et al. found that SUMOylation of Bhlhe40 enhances its repressive potential [30]. In the study reported here, it was seen that SNHG1 promoted the SUMOylation of Bhlhe40.

More importantly, we have shown that SNHG1 acted as a scaffold structure to allow PIAS3 to bind and interact with Bhlhe40 protein, and then contributed to the SUMOylation and nuclear translocation of Bhlhe40. As expected, mutation K279 to arginine (K279R) abolished the effects of SNHG1 on the nuclear translocation of Bhlhe40 protein. Of course, we did not check the protein stability of Bhlhe40; therefore, further studies are needed. Recent studies have shown that targeting the SUMOylation proteins is a novel and effective approach for the treatment of age-related diseases [31]. A small molecule was identified to increase the SUMOylation of SERCA2a and showed great efficiency in the treatment of heart failure [32]. Therefore, our study demonstrating the essential role of SNHG1 in regulating the SUMOylation of Bhlhe40 in HG-induced HA-VSMCs calcification/senescence.

Finally, we tried to explore the target genes of SNHG1. Microarray analysis and GO analysis showed that SNHG1 potentially regulates the autophagy pathway. By experimental screening and validation, Atg10 was identified as a key downstream target of SNHG1. ATG10 is an autophagic E2-like enzyme that interacts with ATG7 to recruit ATG12 and plays a critical role in autophagosome formation [33]. Autophagy is a clearance pathway that maintains cell and tissue homeostasis during diverse cellular stresses (Kim, Hwang, & Kwon [17]. Dysfunction in autophagy is associated with multiple diseases including diabetic vascular complications [34]. The roles of autophagy in vascular calcification/aging are “double-edged sword.” Autophagy can play a protective role against senescence by removing damaged organelles and defective proteins in normal cells [35]. Paradoxically, excessive autophagy could activate cellular senescence [14]. Using in vitro experiments in this study, we found that SNHG1 overexpression suppressed autophagy signaling, which protects HA-VSMCs from HG-induced calcification/senescence. Importantly, forced expression of Atg10 abrogated SNHG1 overexpression-attenuated HA-VSMCs calcification/senescence in HG condition. One potential explanation is that autophagy generates a high flux of recycled products, which are subsequently used for synthesis of the senescence-associated secretory phenotype (SASP) factors and contributing to cellular senescence [14]. Taken together, our work indicates that autophagy is the mechanism underlying SNHG1’s involvement in HG-induced HA-VSMCs calcification/senescence.

It has been proven that Bhlhe40 exerts its inhibitory functions mainly through binding to target gene promoters containing the E-box hexanucleotide sequence (CACGTG) in the nucleus (Kiss, Mudryj, & Ghosh [17]. In our present study, the ChIP and luciferase assay discovered that Bhlhe40 binds to E-box in the Atg10 promoter,indeed, knocking out Bhlhe40 decreases the expression of Atg10. Thus, we speculated that SNHG1 regulates Atg10 expression via Bhlhe40.

However, there are certain limitations to the present study, including the lack of in vivo experiments. Moreover, SNHG1 is commonly described as an oncogenic lncRNA overexpressed in many cancers, including colorectal, liver, lung and prostate cancers (Thin, Tu, & Raveendran [23]. Overexpression of SNHG1 may alleviate diabetic vascular calcification/aging, but may also contribute to tumorigenesis. Therefore, VSMCs-specific expression of SNHG1 may represent a therapeutic approach for diabetic vascular calcification/aging. It will be important in future studies to investigate whether overexpression of SNHG1 in VSMCs has an effect on tumorigenesis in vivo.

## Conclusions

**Fig. 7:**
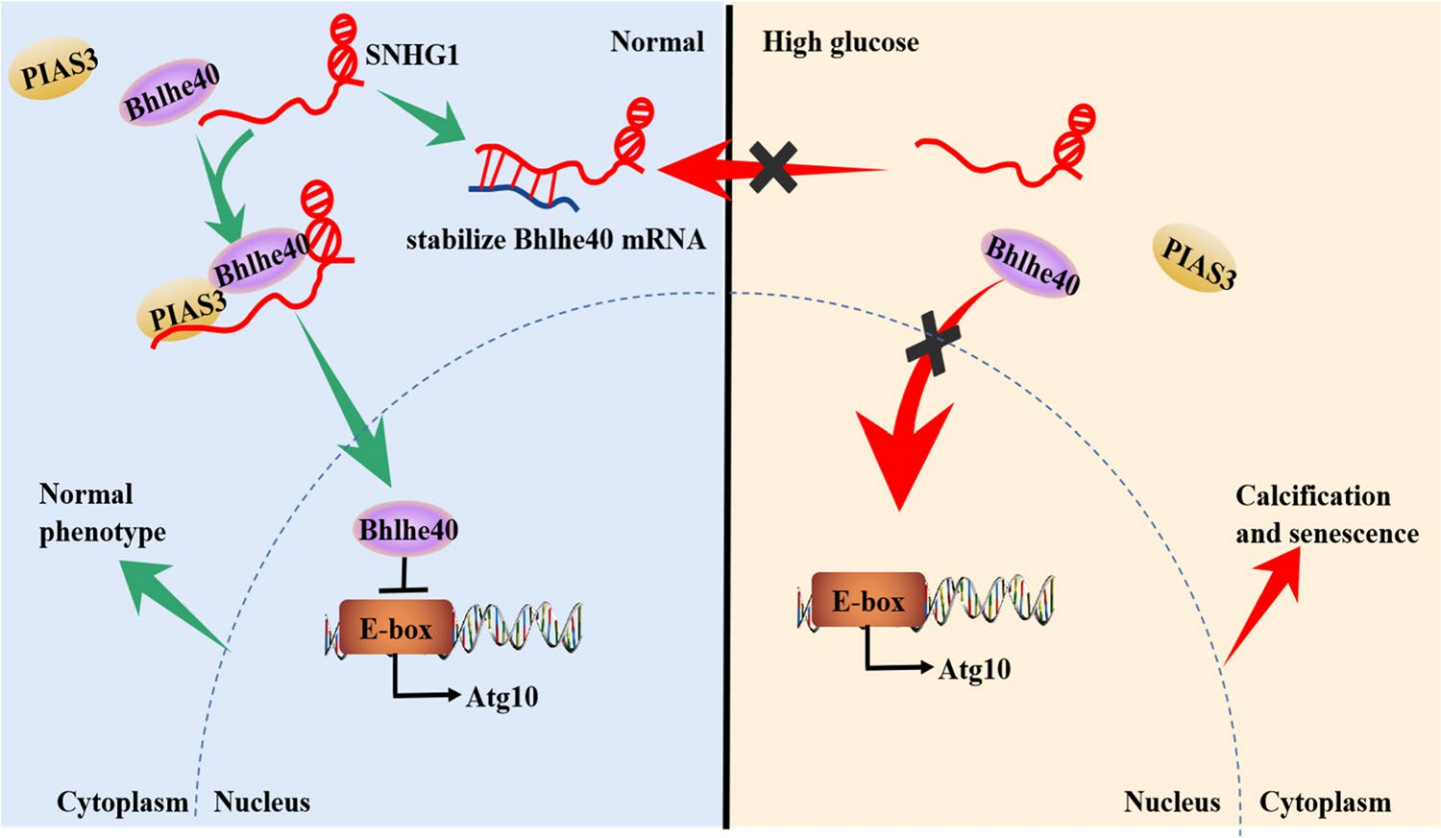
A model for the regulatory mechanisms of SNHG1 in HG-induced HA-VSMCs calcification/senescence

In summary, the present study revealed that SNHG1 is a critical regulator of diabetic vascular calcification/aging. SNHG1 exerts its beneficial effect through two different ways. One is that SNHG1 upregulates Bhlhe40 mRNA expression by forming an RNA-RNA duplex with Bhlhe40 mRNA thereby reinforcing Bhlhe40 mRNA stability. The other one is that SNHG1 facilitates the nuclear translocation of Bhlhe40 protein through enhancing Bhlhe40 SUMOylation (Fig. 7). A full elucidation of the precise mechanisms of SNHG1 in regulating HG-induced HA-VSMCs calcification/senescence will not only increase our knowledge of SNHG1 but also enable the development of an effective therapeutic strategy to treat diabetic vascular calcification/aging.

## Supplementary Information

Below is the link to the electronic supplementary material.

Supplementary file1 (DOCX 76 KB)

## Data Availability

All data and materials are available upon request.
