# Supplementary material for: Long noncoding RNA SNHG1 alleviates high glucose-induced vascular smooth muscle cells calcification/senescence by post-transcriptionally regulating Bhlhe40 and autophagy via Atg10
Source: J Physiol Biochem. 2022 Oct 4;79(1):83–105. doi: 10.1007/s13105-022-00924-2 (PMC9905201; doi:10.1007/s13105-022-00924-2)
Supplement: Supplementary file 1 — Supplementary file1 (DOCX 76 KB) [file 13105_2022_924_MOESM1_ESM.docx]

**Supplementary Table 1. The nucleotide sequence of siRNAs**

| Name | Sequence |
| --- | --- |
| si-SNHG1-1 | Sense: 5’-CCAGCAUCUCAUAAUCUAUTT-3’  Antisense: 5’-AUAGAUUAUGAGAUGCUGGTT-3’ |
| si-SNHG1-2 | Sense: 5’-CCUUCUCUCUAAAGCCCAATT-3’  Antisense: 5’-UUGGGCUUUAGAGAGAAGGTT-3’ |
| si-Bhlhe40-1 | Sense: 5’-CAUGUGAAAGCACUAACAATT-3’  Antisense: 5’-GUUUGUUAGUGCUUUCAGATT-3’ |
| si-Bhlhe40-2 | Sense: 5’-UUCGGAUGAUGAAGGCCAUTT-3’  Antisense: 5’-AUGGCCUUCAUCAUCCGAATT-3’ |
| si-Bhlhe40-3 | Sense: 5’-CGUAGUGAUUAGCUUACUATT-3’  Antisense: 5’-UAGUAAGCUAAUCACUACGTT-3’ |

**Supplementary Table 2. Nucleotide sequences of primers used in the research**

| Gene | Primer sequence (5’ to 3’) |
| --- | --- |
| SNHG1 | Forward: GCCAGCACCTTCTCTCTAAAGC  Reverse: GTCCTCCAAGACAGATTCCATTTT |
| CYP1B1-AS1 | Forward: GTGCAGTTGTGAAGTCAGCA  Reverse: ATGAGCATGGAGAAGGGAGG |
| LINC01356 | Forward : TTAATGGCTGGAGTGCAGTG  Reverse: GCGTTGCTTATGGTCTGACA |
| Bhlhe40 | Forward : ATCCAGCGGACTTTCGCTC  Reverse: TAATTGCGCCGATCCTTTCTC |
| Atg10 | Forward : CCCTTGGATGATTGTGAAGTGA  Reverse: CTGTAGCAGTCGCATCTTATAGC |
| Primers U1 | Forward : GGGAGATACCATGATCACGAAGGT  Reverse: CCACAAATTATGCAGTCGAGTTTCCC |
| 18S | Forward : CAGCCACCCGAGATTGAGCA  Reverse: TAGTAGCGACGGGCGGTGTG |
| β-actin | Forward: ACCCTGAAGTACCCCATCGAG  Reverse: AGCACAGCCTGGATAGCAAC |

**Supplementary Table 3. Nucleotide sequences of ChIP primers used in the research**

| Gene | Primer sequence (5’ to 3’) |
| --- | --- |
| GAPDH | Forward: TACTAGCGGTTTTACGGGCG  Reverse: TCGAACAGGAGGAGCAGAGAGCGA |
| Atg10 | Forward: GTCACCAACACAATCACGGT  Reverse: AGGTACACGGGACTCTAGGT |

**Figure S1**

1.
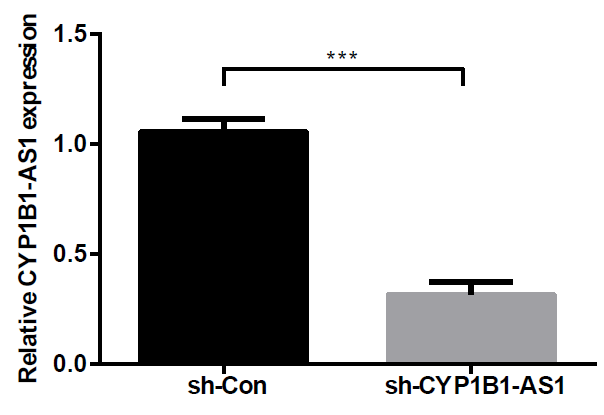

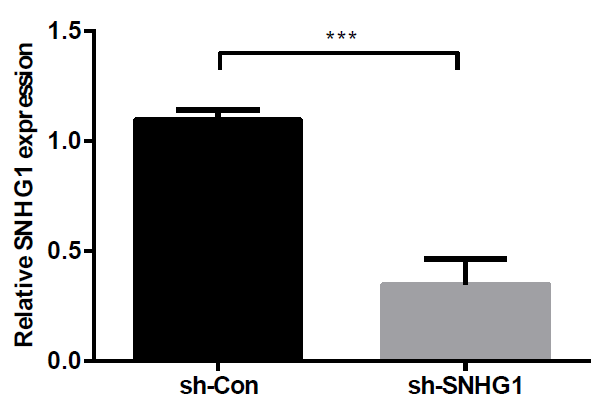
 **B.**


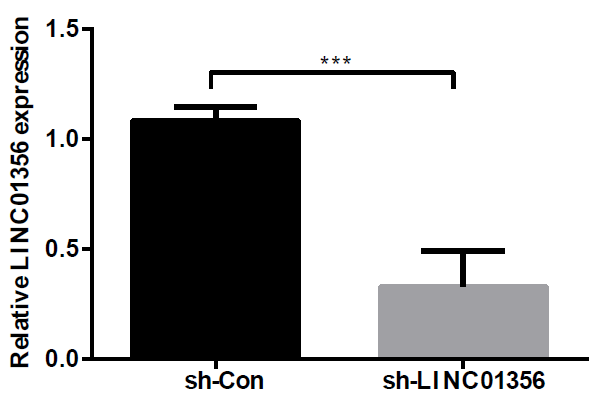
**C.**

**Fig. S1** Transfection efficiency of three si-RNAs by qRT-PCR in HA-VSMCs. **A** SNHG1 **B** CYP1B1-AS1 **C** LINC01356. Results shown are means ± SD from triplicate experiments. ^***^, *P* < 0.001.
